# A Potential Interaction Between Bisphosphonates and Osseointegration of Bone-Anchored Hearing Aid Implants Leading to Late Device Extrusion

**DOI:** 10.7759/cureus.27436

**Published:** 2022-07-29

**Authors:** Jeremy A Mock, Jena Patel, Arun Gadre, Scott Greene

**Affiliations:** 1 Otolaryngology/Head and Neck Surgery, Geisinger Medical Center, Danville, USA; 2 Otolaryngology, Geisinger Commonwealth School of Medicine, Danville, USA; 3 Otolaryngology, Geisinger Medical Center, Danville, USA

**Keywords:** implant extrusion, osteoporosis, bisphosphonate therapy, bone anchored hearing aid, osseointegrated implant

## Abstract

Bisphosphonate therapy is commonly used to treat patients suffering from osteoporosis due to its clinical effectiveness and its generally benign safety profile; however, osteonecrosis of the jaw is a rare side effect that can occur in some patients. A far less elucidated area of concern is the effect of these medications on osseointegrated implants, which require adequate bone formation to ensure long-term viability of the implant. To date, there are no reports in the otolaryngology literature examining the interplay between osteoporosis, bisphosphonate therapy, and osseointegrated bone-anchored hearing aids (BAHA).

In this case report, we describe an osteoporotic patient on bisphosphonates experiencing late bilateral failure of her osseointegrated BAHA implants shortly after starting therapy. Certainly, direct causality cannot be determined from this single report, but the temporal relationship described in this case suggests a potential interaction between bisphosphonate use and delayed failure of the osseointegrated hearing devices. Consequently, otolaryngologists who implant osseointegrated hearing devices should consider offering preoperative counseling to patients receiving bisphosphonate therapy.

## Introduction

Osteoporosis is a common clinical finding with significant deleterious consequences. Most frequently occurring in post-menopausal women, osteoporotic patients are prone to hip and vertebral fractures, which may greatly affect their quality of life and overall mortality. Bisphosphonates (BP) inhibit osteoclastic bone resorption and are often the first-line treatment for osteoporosis. These medications have a long history of success and carry a generally benign safety profile. BPs (specifically in the IV formulations) are also utilized for the treatment of bone disorders including Paget’s disease and multiple myeloma; these particular patient populations have been shown to suffer from one of the more recognizable side effects of BPs, osteonecrosis of the jaw (ONJ). A far less elucidated area of concern is the effect of these medications on osseointegrated implants, which require adequate bone formation to ensure long-term fidelity of the implant. A few reports on this topic can be found in the dental and oral surgery literature in relation to dental implants, but there are no reports in the otolaryngology literature examining the interplay between osteoporosis, BP therapy, and osseointegrated bone-anchored hearing devices (BAHD).

By way of this report, we introduce a case in which the initiation of BP therapy serves as the most probable cause for the failure of bilateral osseointegrated BAHD implants. Certainly, direct causality cannot be determined from this single report, but the temporal relationship described herein suggests a potential interaction between BP use and delayed failure of the osseointegrated hearing devices. Therefore, otolaryngologists who implant osseointegrated hearing devices should consider offering preoperative counseling to patients that are receiving bisphosphonate therapy for osteoporosis, in addition to any other therapies that can alter bone metabolism.

## Case presentation

**Figure 1:**
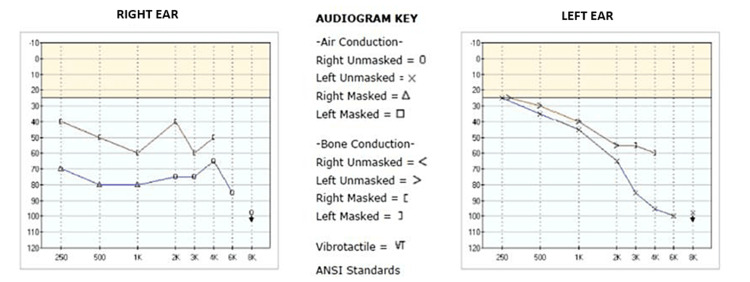
Audiogram pre-extrusion on September 26, 2006

**Figure 2:**
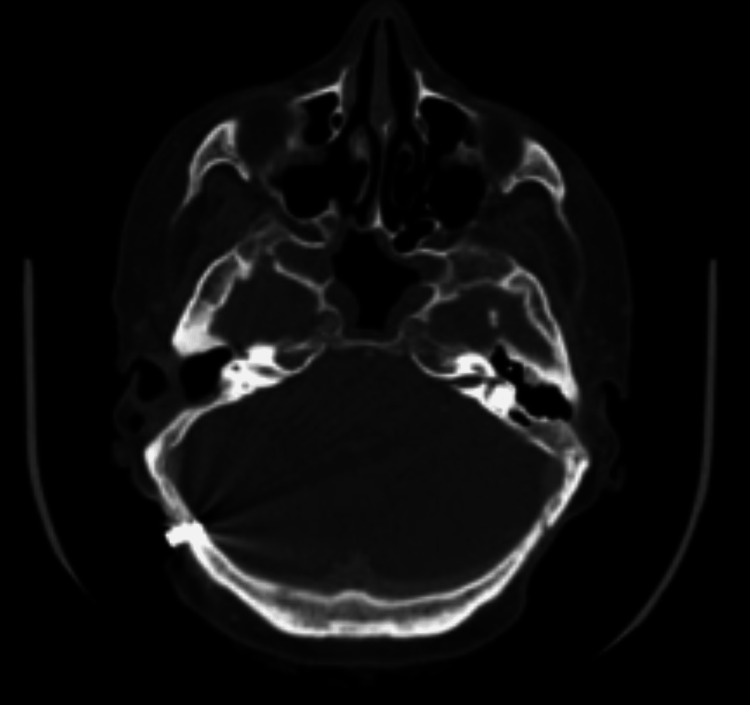
Right BAHA implant visualized on CT scan prior to bisphosphonate therapy on June 23, 2010 BAHA: Bone-anchored hearing aids.

**Figure 3:**
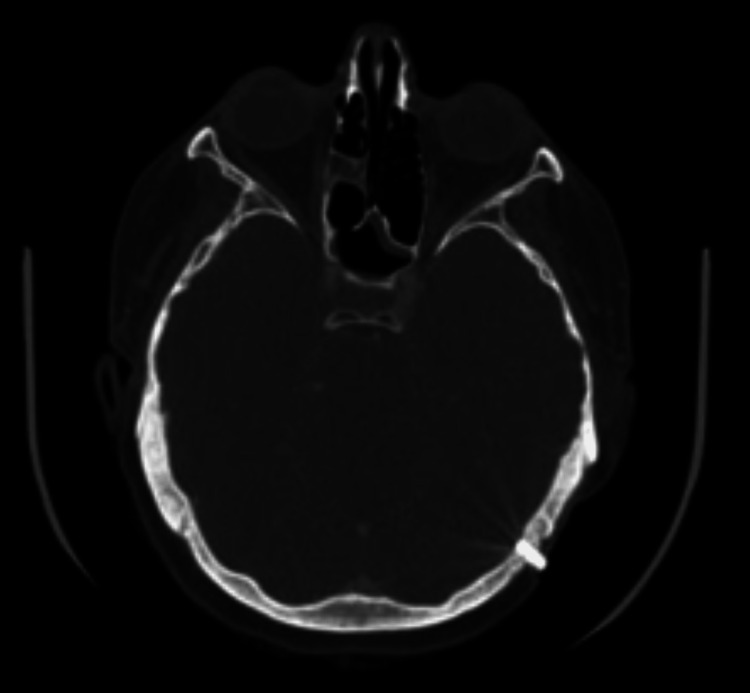
Left BAHA implant visualized on CT scan prior to bisphosphonate therapy on June 23, 2010 BAHA: Bone-anchored hearing aids.

A 52-year-old female presented to the Geisinger Medical Center (GMC) ENT department after extrusion of her left BAHA implant. The patient had an extensive ENT history including bilateral sensorineural hearing loss secondary to measles at age 5, radiation therapy in the 1960s for chronic otitis media, chronic mastoiditis resulting in bilateral mastoidectomies (four surgeries on the right mastoid with the external canal wall down and two surgeries on the left mastoid with an intact canal wall), and bilateral BAHD implants (BAHA Bone Conduction Implants by Cochlear, Sydney, Australia) placed at an outside hospital in March of 2000 and September of 2001. In September of 2006, she transferred her care to GMC, and upon initial evaluation, she had no complaints regarding her osseointegrated implants. The patient expressed that her hearing devices were performing well, and a physical exam found no signs of surrounding soft tissue breakdown or implant instability. For well over a decade, she attended regularly scheduled implant evaluations with unremarkable findings, and routine audiology exams demonstrated unchanged patterns of hearing loss (Figure 1). As such, there is ample documentation of the implants’ stability throughout this time. A CT scan performed in 2010 for an unrelated neck mass (reactive adenopathy) provided visual confirmation of well-integrated implants bilaterally (Figures 2, 3).

**Figure 4:**
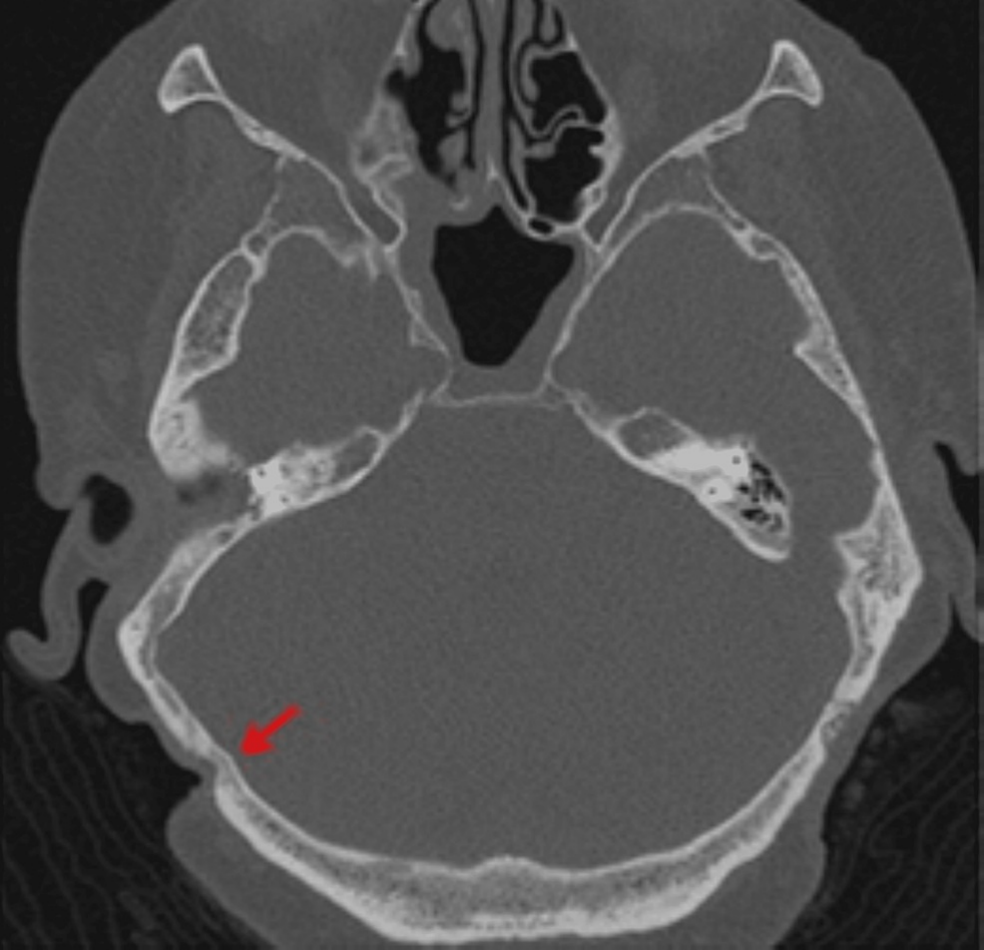
CT scan showing bony changes in the right occipital bone at the previous BAHA site one year after extrusion on November 23, 2013 BAHA: Bone-anchored hearing aids.

**Figure 5:**
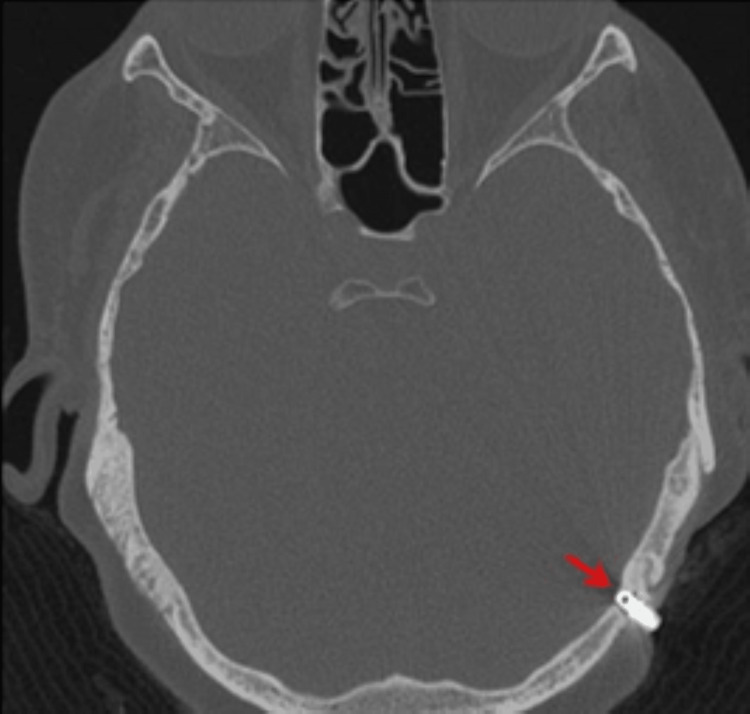
CT scan showing bone loss at the left implant site one month prior to extrusion on November 23, 2013

Due to post-menopausal estrogen deficiency and rib fractures sustained in 2012, a dual-energy x-ray absorptiometry (DEXA) scan was performed. The scan found the patient to be at a high fracture risk based on a T-score of -2.6. Fosamax (Alendronate Sodium) was initiated on July 28, 2012. Dosing was 10 mg per day, which is the standard for patients with osteoporosis. Four months after the start of BP therapy, she was seen at an outside hospital for extrusion of her right implant (which had been stable since implantation 11 years prior). She continued Fosamax for another three months, after which she discontinued the medication due to financial circumstances. One year after extrusion of the right implant and one month after restarting Fosamax (November 20, 2013), the patient was seen by the GMC ENT department for the failure of the left implant (which had been stable since implantation 12 years prior). On exam, the patient had tenderness and mild surrounding erythema at the site of extrusion. A CT scan performed at an outside hospital prior to failure of the left implant was deemed normal; however, when compared to previous scans, the imaging suggested bone loss around the left implant and bony changes at the site of the extruded right implant (Figures 4, 5). The patient was subsequently treated with antibiotics. Additionally, the patient followed recommendations to forego surgical replacement of the hearing devices in light of the previous failures and her need to continue BP therapy. Traditional hearing amplification was provided for her left ear, and she resumed regular mastoid care with no further complications.

Of note, this patient had exposure to additional substances that could have led to abnormal osseous metabolism including tobacco, proton pump inhibitor therapy, and intermittent corticosteroid use; however, the temporal relationship between the initiation of BP therapy and subsequent device failures strongly suggests that BP therapy was a significant contributing factor.

## Discussion

This report highlights a novel case in which BP use was a very likely contributor to the late extrusions of bilateral osseointegrated BAHD implants. A BAHD implant consists of a permanent titanium fixture that is placed in the skull behind the ear and a small detachable sound processor that clips onto the fixture [1]. They are indicated for patients with conductive hearing loss, mixed hearing loss, or unilateral sensorineural hearing loss. Implant success depends on the concept of osseointegration for long-term implant stability. The most common postoperative causes for device failure are local soft tissue reaction or infection [2,3]. Osseointegration in regard to any implant refers to the direct structural and functional connection between ordered, living bone and the surface of an implant. Clinically, osseointegration has been achieved when there is no relative movement between the implanted device and the surrounding bone, and there are no signs of surrounding tissue response toward the foreign body [4]. Successful osseointegration depends on the presence of ample vascularization and adequate osteogenic potential for normal bone remodeling to occur [1]. Therefore, any affront to normal bone metabolism and subsequent bone density represents a theoretical threat to the fidelity of an implant.

Osteoporosis is a systemic disorder characterized by a loss of bone tissue, disruption of bone architecture, and an increase in bone fragility leading to a higher risk of fracture, especially in post-menopausal women. It is diagnosed with a DEXA scan when there is a decrease in the bone density of greater than 2.5 standard deviations below normal. Losses in bone density and architectural integrity are clinically silent until fractures occur [5]. BPs are normally the first-line treatment for osteoporosis and have become increasingly prevalent in recent years due to their relatively benign safety profile. BPs are stable analogs of pyrophosphate and inhibit osteoclast activity through direct incorporation into sites of active bone remodeling. Randomized placebo-controlled trials of various classes of BPs revealed that they generally increase bone mineral density and reduce the risk of vertebral fracture by 30%-50% [6]. Of note, osteonecrosis of the jaw (ONJ) is a rare complication of BP therapy occurring at a reported rate of one in 10,000 patients on oral BPs and an incidence closer to one to 10 per 100 patients on IV preparations [7]. The development of ONJ is multifactorial and has been hypothesized to develop from an over suppression of osteoclasts impairing normal bone remodeling in response to trauma; most commonly, this effect is seen in the mandible where the teeth are routinely subjected to mechanical stress [8,9]. Osteonecrosis of the jaw has been documented at even higher rates in patients on BP therapy undergoing dental implant surgery [10].

There are no available reports in the otolaryngology literature describing the failure of osseointegrated hearing devices in relation to BP use, and there is only a small amount of evidence exploring this interaction in the dental and oral surgery literature. For example, Zahid et al. completed a retrospective radiographic study of 362 patients and found 26 dental implant patients using BPs (a total of 51 dental implants). The authors found a statistically significant relationship between BP therapy and implant thread exposure, an expected precursor to implant failure (P = 0.001; OR = 3.25) [11]. Notably, intravenous BP use is considered an absolute contraindication to the placement of dental implants [12].

Most often, postoperative complications after the placement of BAHD occur due to failure of osseointegration, trauma, infection, or fixture dislodgement, with no mention of BP use [13,14]. A 2013 meta-analysis by Kiringoda et al. found the quality of existing studies reporting the incidence of complications after osseointegrated hearing aid surgery to be of poor quality and to lack uniformity. However, based on the available data, the authors concluded that the majority of postoperative complications were minor. Specifically, this review included 2,134 patients who underwent a total of 2,310 implants; the failure of osseointegration ranged from 0% to 18% in adults and mixed populations (adult and pediatric patients), and the total rate of implant loss, including delayed device failure, ranged from 1.6% to 17.4% [14]. The review ultimately determined that the implantation of these devices is safe and worth the expected benefit to the quality of life despite the potential risks.

## Conclusions

As such, no previous description of the relationship between osteoporosis, BP use, and osseointegrated hearing devices has been presented in the otolaryngology literature. Given the growing prevalence of osteoporosis and consequent use of BP therapy, this case highlights the potential concerns for any otolaryngologist considering placement of osseointegrated implants in a patient currently undergoing BP therapy or initiating BP therapy in any patient with an existing implant.
